# Predictive Processing in Sign Languages: A Systematic Review

**DOI:** 10.3389/fpsyg.2022.805792

**Published:** 2022-04-14

**Authors:** Tomislav Radošević, Evie A. Malaia, Marina Milković

**Affiliations:** ^1^Laboratory for Sign Language and Deaf Culture Research, Faculty of Education and Rehabilitation Sciences, University of Zagreb, Zagreb, Croatia; ^2^Laboratory for Neuroscience of Dynamic Cognition, Department of Communicative Disorders, College of Arts and Sciences, University of Alabama, Tuscaloosa, AL, United States

**Keywords:** sign language, systematic review, predictive processing, linguistic prediction, cognitive neuroscience

## Abstract

The objective of this article was to review existing research to assess the evidence for predictive processing (PP) in sign language, the conditions under which it occurs, and the effects of language mastery (sign language as a first language, sign language as a second language, bimodal bilingualism) on the neural bases of PP. This review followed the Preferred Reporting Items for Systematic Reviews and Meta-Analyses (PRISMA) framework. We searched peer-reviewed electronic databases (SCOPUS, Web of Science, PubMed, ScienceDirect, and EBSCO host) and gray literature (dissertations in ProQuest). We also searched the reference lists of records selected for the review and forward citations to identify all relevant publications. We searched for records based on five criteria (original work, peer-reviewed, published in English, research topic related to PP or neural entrainment, and human sign language processing). To reduce the risk of bias, the remaining two authors with expertise in sign language processing and a variety of research methods reviewed the results. Disagreements were resolved through extensive discussion. In the final review, 7 records were included, of which 5 were published articles and 2 were dissertations. The reviewed records provide evidence for PP in signing populations, although the underlying mechanism in the visual modality is not clear. The reviewed studies addressed the motor simulation proposals, neural basis of PP, as well as the development of PP. All studies used dynamic sign stimuli. Most of the studies focused on semantic prediction. The question of the mechanism for the interaction between one’s sign language competence (L1 vs. L2 vs. bimodal bilingual) and PP in the manual-visual modality remains unclear, primarily due to the scarcity of participants with varying degrees of language dominance. There is a paucity of evidence for PP in sign languages, especially for frequency-based, phonetic (articulatory), and syntactic prediction. However, studies published to date indicate that Deaf native/native-like L1 signers predict linguistic information during sign language processing, suggesting that PP is an amodal property of language processing.

**Systematic Review Registration:**

[https://www.crd.york.ac.uk/prospero/display_record.php?ID=CRD42021238911], identifier [CRD42021238911].

## Predictive Brain

Our understanding of the human brain as a source of cognition has historically focused on the brain as generating a response to external stimuli. Recently, there has been a paradigm shift in the field of cognitive neuroscience. The traditional concept of the brain as a passive, bottom-up receiver of external information has been replaced by the notion of the brain as an active predictor of the environment, generally termed as predictive processing (PP). The main idea behind the PP is that “the brain is a sophisticated hypothesis-testing mechanism, which is constantly involved in minimizing the error of its predictions of the sensory input it receives from the world” (Hohwy, 2013, p. 1).

In the last decade, the notion of PP has gained wide recognition as a model of cognitive processing applied to a variety of brain functions (Friston, 2010; Clark, 2013; Hohwy, 2013; Chanes and Barrett, 2020; Ficco et al., 2021; Perrinet, 2021), including language production and comprehension (Federmeier, 2007; Altmann and Mirković, 2009; Huettig, 2015; Lewis and Bastiaansen, 2015; Kuperberg and Jaeger, 2016; Ferreira and Qiu, 2021). Several aspects of the mechanism of PP have attracted the attention of researchers seeking to specify the model in more detail. These include the modality-dependent structure of hierarchical predictions and the interplay between prediction errors at various levels of linguistic processing (e.g., syntax vs. semantics).

### Predictive Processing and Language

Most studies that addressed PP in language comprehension used the visual modality (i.e., reading) to assess PP in spoken language processing (e.g., Szewczyk and Schriefers, 2013; Bonhage et al., 2015; Wlotko and Federmeier, 2015; Rommers et al., 2017). In general, they report that prediction facilitates language comprehension. In the auditory modality for speech, multiple electrophysiological indicators in time- and frequency domains characterize automatic predictive processing at a range of scales (see Bendixen et al., 2012, for a review). Moreover, studies using simulation/modeling approaches to speech perception (Donhauser and Baillet, 2020) have shown that distinct types of PP (e.g., based on uncertainty vs. surprise metrics for phoneme sequences) elicit responses at different frequencies. This suggests that in human speech signal, PP concurrently proceeds at multiple scales.

The studies that have investigated scale-specific PP in human language from the point of view of specific levels of language structure consistently uncovered predictive processes at the levels of the language studied, e.g., phonology (Donhauser and Baillet, 2020), form and meaning (Freunberger and Roehm, 2016; Ito et al., 2016), or syntax (Yoshida et al., 2013; Bonhage et al., 2015; Droge et al., 2016). Studies at the interface between syntax and semantics, e.g., studies on disambiguation of garden-path sentences (reduced subject and object relative clauses in English), have shown that prediction errors are detected and further predictions refined at the earliest when critical linguistic information (either syntactic or semantic) is available for the language in question. For example, a magnetoencephalographic (MEG) study of Dutch language processing (Lewis et al., 2016) has shown that the difference between subject and object cognates affects neural processing at the position of the auxiliary indicating the grammatical number. Studies on the contribution of verb and noun semantics to the disambiguation of relative clauses in English (Malaia et al., 2009, 2012, 2013; Malaia and Newman, 2015) have shown that participants consistently relied on prior linguistic information (e.g., noun animacy, verbal telicity) when interpreting incoming words in complex sentences. However, participants quickly revised their predictions when they received either new semantic or new syntactic information, depending on what had previously occurred in a given sentence. This group of studies provided important supporting information for rapid error correction across linguistic interfaces.

### Models of Predictive Processing

The concept of PP is not a unitary concept; among the multiple models developed, some aim to describe and predict cognition or decision-making processes in general; others focus on the mechanisms underlying linguistic prediction. The core mechanisms involved in PP, which appear across multiple models, and have been confirmed across multiple experimental studies, include top-down processing, statistical estimation, hierarchical processing, prediction, prediction error minimization, Bayesian inference, and predictive control (for a detailed review see Wiese and Metzinger, 2017). Huettig (2015) proposed a taxonomy of PP models based on (1) the type of data the models aim to explain and predict, and (2) the mechanisms (cognitive or neural) purported for the model, arriving at four broad groups.

The first group of models (Kuperberg, 2007; Kahneman, 2011) with general domain of application (cognition or language) assumes two different mechanisms (systems) involved. The first system (“thinking fast”) relies on rapid re-activation of prior knowledge based on incoming information; the second system relies on conscious allocation of cognitive resources optimized for the task at hand (i.e., “thinking slow”) (Kuperberg, 2007; Kahneman, 2011). Secondly, there is a group of models that claim that both linguistic and non-linguistic PP rely on the same predictive mechanisms (Altmann and Mirković, 2009), and that linguistic prediction relies on event knowledge (Metusalem et al., 2012).

Another group of PP models is grounded in production-based approaches to predictive processing. For example, Pickering and Garrod (2007, 2013) suggest that production systems facilitate language comprehension via forward models. Specifically, they argue that the comprehender performs a covert imitation, which is realized as a motor simulation of the speaker’s utterances. Dogge et al. (2019) proposed Hybrid Prediction Model that, in addition to motor forward modeling, includes predictive coding that does not rely on efference copies of the motor simulation. Another production-based model of PP is the PARLO (Production Affects Reception in Left Only) framework proposed by Federmeier (2007). According to PARLO model, the left hemisphere is more prone to top-down processing; and, since the areas for language comprehension and production are predominantly found in the left hemisphere, this results in strong feedback connectivity in support of PP.

Huettig (2015) also proposes a multiple-mechanism model for linguistic PP, named PACS (production-, association-, combinatorial-, simulation-based prediction). Huettig suggests that, given the complexity of the PP phenomenon, multiple mechanisms may be involved in predictive processing, depending on the task and/or user experience in specific context and interact with each other. For example, comprehenders might use fully specified production representations for producing a predictive model or refine the model using simple associative mechanisms. The combinatorial component of the model emphasizes the interaction of multiple linguistic constraints that influence linguistic prediction. Lastly, Huettig (2015) suggests event simulation as a possible, but not necessary, element of PP.

Another model involving multiple mechanisms is the Multiscale Information Transfer framework (MSIT, Blumenthal-Dramé and Malaia, 2019). This model assumes parallel processing of incoming linear signal at multiple temporal scales (and thus, by multiple mechanisms). As the incoming sensory-linguistic signal is parsed into units at multiple scales (e.g., as syllables, words, and clauses), each level of linguistic processing (phonotactic, semantic, syntactic, pragmatic) quickly provides and discards predictions based on sequence probabilities (syntagmatic) and linguistic structure availability (paradigmatic), under the top-down guidance of the processor’s sentence- and discourse-level predictions, thus allowing for both feed-forward and feedback effects.

Most of the linguistic models for PP mentioned above are based on research in spoken languages, with a focus on auditory modality, and, computationally, dealing with one-dimensional timeseries data. The study of sign languages (SLs), thus, can be informative for PP for several reasons. First, SLs are natural languages realized in the visual modality, i.e., reliant on 3D or 2D (video-type) processing. Therefore, SLs provide a unique opportunity to shed light on the underlying interplay of vision and cognitive processes in relation to the temporal structure of linguistic prediction. Second, examination of how linguistic PP unfolds temporally in the visual domain (as opposed to reading printed text, which is visible all the time) can contribute to refining existing PP models by identifying at which linguistic levels and/or interfaces PP occurs. Third, by examining PP in SLs as compared to PP in spoken languages modality-specific effects on PP can be isolated from modality-independent components of PP. The latter would emphasize linguistic and cognitive universals across sign and spoken languages with respect to PP.

Our goal in this work is to systematically assess the evidence for PP in SLs, the task and stimulus conditions under which it has been documented, the effects of individual differences in predictive processing (e.g., SL competence or age of SL acquisition) on the neural bases of PP, and identify the gaps in research which would allow for best possible contribution to modeling PP in human languages. We also aim to evaluate the effects of the physical and linguistic parameters of SL(s) on the PP phenomenon and to set the stage for careful experimental work in the future.

## Methods

### Systematic Review Protocol

This systematic review was conducted in compliance with Preferred Reporting Items for Systematic Reviews and Meta-Analysis (PRISMA; Page et al., 2021). It was pre-registered in the PROSPERO registry (Radošević et al., 2021;; registration number CRD42021238911) to reduce the risk of bias that might occur during the review process. We defined our search and eligibility criteria according to the PICOS model (populations, interventions, comparators, outcomes, and study design).

#### Populations

In terms of participant-related variables, we defined the following inclusion criteria: studies on SL processing of all linguistic proficiency profiles given the probability of different processing mechanisms (see Krebs et al., 2021): proficiency in SL as a first language (L1) in Deaf signers or Children of Deaf Adults (CODAs), or SL as a second language (L2), i.e., L2 learners across age ranges. To select only research that focused on PP in SLs, we applied the following exclusion criteria: Studies of SL processing that focused exclusively on non-signers; non-human sign language processing (artificial intelligence and machine learning studies or brain-computer interface), and animal models.

#### Intervention

Our review focuses on SL processing at any linguistic level. Since defining language processing at a particular level does not add value to the search, we omitted it from our queries.

#### Comparison

With respect to studies of SL processing, comparators would be other visual processing (non-sign-language-based) or differences in processing within linguistic levels. Because one of the two controls is certain to be present in each SL study, Addition of any terms for comparisons did not add value to the search.

#### Outcomes

In our review, outcome is defined as evidence of predictive processing or entrainment during SL processing. As such, it was included in the search.

#### Study Design

Our goal was to search for any and all research-based evidence of PP in SLs. Therefore, defining the study design would not add value to the search. However, the record had to be original research, i.e., review articles were not included. As for the status of the records, they had to have been published in a peer-reviewed outlet. Therefore, only articles published in peer-reviewed journals, conference proceedings, doctoral dissertations published in digital repositories, and book chapters were considered. Finally, the record had to be written in English.

### Data Sources and Search Strategy

**FIGURE 1:**
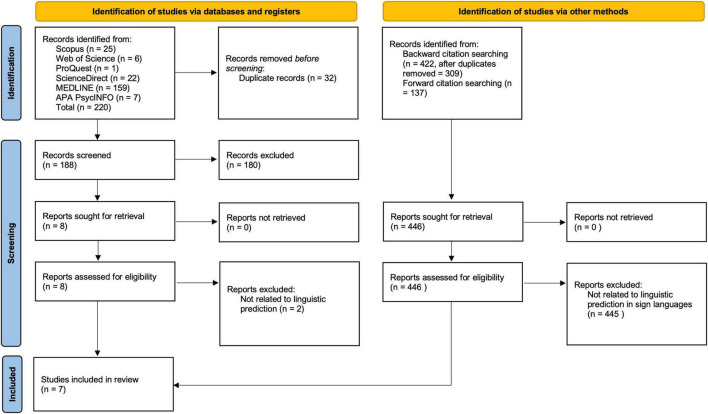
PRISMA 2020 flow diagram (adapted from Page et al., 2021).

We searched the Scopus, Web of Science, ScienceDirect, and ProQuest databases. In addition, the database APA PsycInfo was searched through the EBSCOhost platform and the MEDLINE database was searched through PubMed. The search strings we used for the search can be seen in Appendix 1. All sources were searched regardless of the year of publication. We conducted the initial search in March 2021, followed by several re-runs, with the final one in July 2021. In addition, we performed a citation search of studies selected to include in the review after full-text examination (see PRISMA flow diagram, Figure 1). Based on the final number of records, we performed a backward citation search, i.e., we screened all references cited in studies that had passed the full-text eligibility assessment. To ensure that no recent, potentially relevant studies were missed in the database search, we also performed a forward search. We searched Scopus, Web of Science, and ProQuest in July 2021 for new studies citing the same studies that had passed the full-text eligibility assessment.

### Study Selection and Data Extraction

At the identification level, the first author searched for records in databases and screened the retrieved records at the title and abstract level. Subsequently, all three authors independently reviewed the full texts. Disagreements were resolved in a detailed discussion. The first author then performed the backward and forward citation search, as described in section “Data Sources and Search Strategy,” analyzed each record selected for systematic review and recorded the targeted data in a Microsoft Excel spreadsheet, based on the variables from section “Systematic Review Protocol.”

Four groups of variables to seek for were established: type of stimuli, task, target language in the experiment, and participants’ SL dominance. First, the type of stimuli aims to distinguish between dynamic and static SL stimuli, or in the case of spoken language stimuli—printed words on the screen or auditory presented stimuli for hearing participants. Second, the type of task refers to the paradigm used, from which the tasks are derived. Third, the target language in the experiments aims to separate SL, spoken language and written language. Finally, participants’ SL dominance refers to SL as L1, L2, or whether participants were bimodal bilinguals, either Deaf or hearing.

Although this review is not clinical, we have identified a bias in the selection. Namely, only records in which findings were discussed from the perspective of PP were included. Given the wealth of evidence for PP in language, we assume that some previous studies may have PP underlying their results. However, because they were not discussed from this perspective, they did not meet the inclusion criteria.

## Results

### Study Selection and Characteristics

The first author retrieved and screened 220 records to exclude those that did not meet the inclusion criteria. After duplicates were removed, 188 records were screened at the title and abstract level (see PRISMA flow diagram, Figure 1). After excluding 180 records (artificial intelligence studies, brain-computer interface, spoken language studies, etc.), 8 full-text records were assessed for eligibility. During the review of the full texts, a further 2 records were excluded as they did not meet the inclusion criteria. Namely, they were not related to linguistic prediction. At this stage, the results were independently reviewed by the remaining two authors. There was no disagreement.

Backward citation screening yielded 422 references (309 after duplicates were removed). Forward citation screening yielded 137 references as follows: in SCOPUS Brookshire et al. (2017) were cited 15 times, but these studies were not related to PP in SLs. In Web of Science, they were cited in 18 articles but had no reference to PP in SLs, the same with 4 citations in ProQuest. Citations of Brookshire (2018) were not found in Scopus, Web of Science, or ProQuest. Brozdowski and Emmorey (2020) were cited once in Scopus and once in Web of Science, but this work was not related to PP in SLs. Brozdowski (2018) was not cited in Scopus, Web of Science, or ProQuest. Hosemann et al. (2013) was cited 32 times in Scopus, 28 times in Web of Science, and 22 times in ProQuest. No new references were relevant to this review. Lieberman et al. (2018) were cited 8 times in Web of Science and 8 times in Scopus. Only one paper from Scopus met the inclusion criteria, Wienholz and Lieberman (2019).

Records that appeared to meet the criteria, but were excluded after full-text evaluation are Bosworth et al. (2019) and Kubicek and Quandt (2019). Bosworth et al. (2019) passed the screening on the title and abstract level because they examined the visual properties of American Sign Language (ASL) and there was a possibility that they discussed these visual cues in the context of prediction. However, after the full-text evaluation, it turned out this was not the case. On the other hand, Kubicek and Quandt (2019) investigated the activation of sensorimotor systems, i.e., the action observation network, while Deaf signers and hearing non-signers perceived one-handed or two-handed signs. Compared to Deaf signers, hearing non-signers showed greater activation of the sensorimotor cortex as measured by EEG desynchronization. They also found that the sensorimotor cortex was sensitive to one-handed and two-handed signs in both groups, but they activated the mirror system only in the Deaf group. However, these results were not related to (production accounts of) linguistic prediction (cf. Pickering and Garrod, 2007; Pickering and Gambi, 2018), so this work was excluded from further review. Thus, a total of 7 publications were included in the final review, of which 5 were published articles and 2 were doctoral dissertations.

The small number of records remaining may be due to several reasons. First, SL sentences have only been used in processing experiments in the last decade (first by Capek et al., 2009, followed by Hosemann et al., 2013; Hänel-Faulhaber et al., 2014, etc.). However, the degree to which these sentences were natural is questionable given that the persons recording the stimuli were advised to reduce non-manual markings (Hosemann et al., 2013; Hänel-Faulhaber et al., 2014). This is important because transitional movements between signs that have been removed (e.g., Neville et al., 1997) play an important role in providing cues for PP. Second, in general psycholinguistic research on SLs, i.e., not only in the context of PP, it is important to control for psycholinguistic variables such as word frequency, cloze probability, and neighborhood density. These metrics are derived from corpora that are still being developed for SLs, which may be the reason for the smaller number of PP studies. Currently, such information exists only for ASL (ASL-LEX 2.0, Sehyr et al., 2021). Third, as stated in section “Study Selection and Data Extraction,” there is a possibility of a selection bias. Namely, we assume there might be studies that do have PP underlying their findings. However, if the authors did not focus on PP directly, this study could not meet the inclusion criteria and was probably not retrieved using our search queries.

### Synthesized Findings

The key characteristics of each study included in the final review are presented in Table 1. In the following sections, we summarize the records included in the final review by type of research method.

**TABLE 1:** Key characteristics of the studies inluded in the final review.

Study	Type of study and task	Deaf/Hearing	SL dominance and age of acquisition	Type of language stimuli	Target language	Results
Brookshire (2018) Study 1 and Brookshire et al. (2017)	EEG/watching ASL storytelling	(1) Deaf, (2) Hearing	(1) L1, < 5 (mean 1.1 years); (2) non-signers	Dynamic (Sentences)	ASL	EEG coherence to visual oscillations in sign language in signers (0.4–5Hz; frontal and occipital channels) and non-signers (0.8–3.5Hz; central and occipital channels).
Brozdowski (2018) Experiment 1 and Brozdowski and Emmorey (2020)	Behavioral/manual shadowing	(1) Deaf, (2) Hearing	(1) L1, < 6 (early or native SL exposure); (2) non-signers	Dynamic [Videos of (a) pseudosigns, (b) grooming gestures]	ASL	Evidence of egocentric bias (a proxy to motor simulation) only in non-signers shadowing grooming gestures; no facilitatory effect of familiarity in signers; signers’ productions had more consistent lag times than non-signers’ productions.
Brozdowski (2018) Experiment 2	Behavioral/recognition task	(1) Deaf, (2) Hearing	(1) L1, < 6 (early or native SL exposure); (2) non-signers	Dynamic [Videos of (a) pseudosigns, (b) grooming gestures]	ASL	Signers had significantly slower RTs for shadowing blurred pseudosign handshapes
Hosemann et al. (2013)	EEG/semantic mismatch; acceptability and evaluation judgment	Deaf	L1, native or < 3 years	Dynamic (Sentences)	DGS	Unexpected signs elicited a biphasic N400-late positivity effect. Moreover, N400 onset began during the transitional phase, i.e., before the onset of the critical sign.
Lieberman et al. (2018)	Eye-tracking/visual world; adults clicked on the target, children pointed to it	Deaf	Adults: L1, 9 native, 8 non-native (but L1 for at least 19 years); children: L1, 17 at least 1 Deaf parent, 3 had hearing parents but were exposed to ASL by the age of 2:6	Dynamic (Sentences)	ASL	In semantically constraining sentences both groups made anticipatory gaze to the target picture, appearing before the target noun.
Wienholz and Lieberman (2019)	Eye-tracking/visual world; adults clicked on the target, children pointed to it	Deaf	Adults: L1, 9 native, 9 non-native (but L1 for at least 19 years); children: L1, 17 at least 1 Deaf parent, remaining 3 had hearing parents but were exposed to ASL by the age of 2:6	Dynamic (Sentences)	ASL	Anticipatory looks to a target picture were observed in both groups; the adults made target fixations earlier in the sentence and preferred the adjective-noun order, unlike the children.

#### Eye-Tracking Research

An eye-tracking study of ASL found evidence of semantic prediction (Lieberman et al., 2018). Using the visual world paradigm, they investigated whether linguistic predictions modulate signers’ (adults’ and children’s) focus on linguistic or non-linguistic information in the visual modality. They found that under semantically constrained conditions (e.g., a constraining verb at the beginning of a sentence), both children and adults shift their gaze from the ASL video (linguistic information) to the target image (non-linguistic information). Importantly, these gazes were anticipatory in both groups, i.e., they appeared before the target noun, thus suggesting PP.

Their work was extended by another study (Wienholz and Lieberman, 2019) that investigated how signers (adults and children) allocate their gaze in the visual world paradigm consisting of linguistic and non-linguistic information in ambiguous contexts. Both groups looked anticipatively at the target image when it was possible to disambiguate it. Moreover, both groups made more fixations to the target in adjective-noun sentences than in noun-adjective sentences. However, this occurred earlier in the sentence for the adults and later for the children. This suggests that PP is already developed in the children from this study between the ages of 4:1 and 8:1. However, the temporal distribution of their eye-gaze suggests that they are more influenced by competing linguistic distractors during processing than adults who have fully acquired the language, although ASL was L1 in both groups.

#### Behavioral Research

In addition to the studies that focus on language comprehension, there is also work that focuses on the interface of language production and motor production. Brozdowski (2018) investigated prediction at the phonological level, i.e., linguistic and non-linguistic prediction via forward models, in a total of four experiments. Experiment 1 and 2 are discussed further, while Experiment 3 and 4 were excluded as being out of the scope of this review. Forward models suggest that humans covertly simulate language production as they comprehend the incoming linguistic input. In Experiment 1, also published as Brozdowski and Emmorey (2020), he used shadowing as a proxy to covert imitation, a proposed mechanism underlying motor simulation in forward models. Deaf signers and hearing non-signers had to shadow either pre-recorded videos of themselves, a friend, or a stranger. The shadowed stimuli were either pseudosigns (phonologically plausible in ASL, but semantically empty units, therefore still considered linguistic) or grooming gestures (non-linguistic stimuli). Moreover, pseudosigns and grooming gestures could be either one-handed or two-handed. In this way, egocentric bias and visual familiarity effects could be controlled for, as they may facilitate PP. Controlling for handedness was also important, as suppression of the non-dominant hand may have resulted in longer lag times for one-handed signs. However, only non-signers showed the effect of egocentric bias, but only for the grooming-gesture condition, which is understandable given that they are non-signers and cannot predict ASL phonology from the pseudosign condition. Moreover, signers had slower shadowing production for one-handed signs than for two-handed signs. Based on this data Brozdowski (2018) and Brozdowski and Emmorey (2020) conclude that the results do not provide strong evidence for motor simulation accounts of PP.

In Experiment 2, Brozdowski aimed to further investigate phonologically based prediction during the transitional phases between pseudosigns and grooming gestures. Deaf signers and hearing non-signers were asked to monitor for a specific item while reaction times (RTs) were measured. Stimuli were presented either normally or with blurred handshape in the transition phase, or only the still frame of the last frame before the transition movement was shown. As in Experiment 1, the stimuli were either pseudosigns or one- or two-handed grooming gestures. Only signers had significantly slower RTs for blurred handshapes, and only in the pseudosign condition, suggesting that signers made predictions about upcoming phonological representations. However, one-handed stimuli were easier to predict, which contrasts with the expected suppression of the non-dominant hand during motor simulation in forward models. In sum, given the partially opposite findings, the authors conclude that there is not enough evidence that it is precisely the motor simulation that underlies PP in ASL.

#### EEG Research

A study of German Sign Language (*Deutsche Gebärdensprache*, DGS) also found evidence of semantic prediction. Hosemann et al. (2013) investigated whether production-based forward models of language processing (cf. Pickering and Garrod, 2007) are applicable to the visual modality, namely to DGS. The study used a semantic expectancy mismatch design in which the sentence-final verb in the stimuli sentences could be either an expected or unexpected. EEG was recorded while Deaf native signers watched natural DGS sentences. Analysis of event-related potentials (ERPs) indicates that unexpected signs triggered a biphasic N400 effect with late positivity. Moreover, the N400 onset started during the transition phase between two signs, i.e., before the onset of the critical lexical sign. Hosemann et al. (2013) argue that signers made predictions about upcoming linguistic information via forward models, as they relied on the transitional movements seen before the lexical sign. This work aligns with findings from eye-tracking studies on semantic predictions made by ASL signers (Lieberman et al., 2018; Wienholz and Lieberman, 2019) by confirming the existence of semantic prediction in another SL unrelated to ASL.

Unlike aforementioned studies, which focused more on the content of predictions (i.e., what is predicted), Brookshire (2018) used electroencephalography (EEG) to investigate the temporal aspects of prediction (at what point PP is observed) in two studies. The first study (published as Brookshire et al., 2017) aimed to evaluate whether neural oscillations in the human brain entrain to linguistic input in the visual modality—ASL. Brookshire et al. (2017) quantified the change in visual signal using an Instantaneous Visual Change (IVC) metric that measures the relative pixel value change from frame to frame in stimulus videos, reducing the spatial dimensions in 2D video to a single scalar value in time. This way of calculating the metric acts as a spatial frequency filter whose properties change from frame to frame, depending on the colors and contrast within the scene. For example, a gross motion of a signer’s arm will affect a large number of pixels, resulting in a large IVC value, while rapid complex finger motion will affect a small number of pixels, resulting in a low IVC value. Thus, the IVC metric might contain a small portion of the information inherent in the sign language signal (see Borneman et al., 2018); however, given the spatial nature of sign language, the majority of the information contained in the sign language video recording is lost. Based on this crude metric of visual input, Brookshire et al. (2017) found that signers showed higher coherence in the frequency range of 0.4–5 Hz, peaking at 1 Hz, over frontal and occipital electrodes compared to non-signers exposed to the same stimuli. The group concluded that in signers, increased coherence to gross changes in visual input over the frontal electrodes likely indicates top-down control. Non-signers also showed coherence to the visual input (which was also ASL—no control stimuli were used) in the 0.8–3.5 Hz range over the central and occipital sites. Based on these findings, Brookshire et al. (2017) argued that entrainment to gross (i.e., low-frequency in both temporal and spatial dimensions) variability in the visual signal may be an amodal property of the brain aiming to synchronize to a perceptually prominent modality. However, these conclusions are limited by the confounds in experimental design: lack of control stimuli (i.e., stimuli other than SL) and the crudeness of the visual metric, which does not evaluate information-bearing spatiotemporal frequencies in the sign language signal.

### Effects of Stimuli, Sign Language Proficiency of Participants, and Task Types

All studies included in the final synthesis used dynamic sign stimuli, i.e., videos, for the stimuli under which PP was observed. However, task conditions under which PP was observed varied substantially. Brookshire et al. (2017) and Brookshire (2018), asked Deaf signers and hearing non-signers to watch ASL videos while EEG was recorded, with no explicit behavioral task reported (i.e., in a sense, without a comprehension control). Hosemann et al. (2013) used a semantic mismatch paradigm, recording EEG while Deaf signers looked at signed sentences. After viewing a sentence, participants had to determine whether the sentence was correct or incorrect (acceptability task) and then rate how confident they were in their answer (confidence rating). Brozdowski and Emmorey (2020), as well as Experiment 1 by Brozdowski (2018) used the manual shadowing task, in which Deaf signers and hearing non-signers were asked to repeat pseudosigns and gestures as they watched them. Experiment 2 (Brozdowski, 2018), engaged Deaf signers and hearing non-signers in a recognition task, in which participants had to press a key once they recognized a target from a set of pseudosigns or grooming gestures. The remaining two studies used eye-tracking to examine gaze distribution of Deaf adults and children in the visual world paradigm (Lieberman et al., 2018; Wienholz and Lieberman, 2019). In both studies, adults were asked to click on the target picture, while children pointed with a finger and the experimenter then clicked on the target.

Examined studies did not provide information on whether cross-modal prediction occurred for bilinguals, as almost all studies focused predominantly on ASL, with one study on DGS. Regarding the influence of population parameters such as SL dominance and age of acquisition, the results are less conclusive as the only two groups recruited were either Deaf native or native-like L1 signers or hearing non-signers. However, the developmental course of PP has been investigated by Lieberman et al. (2018) and Wienholz and Lieberman (2019) by examining how adults and children distribute eye-gaze in the visual world paradigm. Their studies suggest that basic semantic prediction is developed in children as young as 4–8 years of age.

## Discussion

Nowadays, predictive processing is recognized as a model of cognitive processing applicable to multiple cognitive domains, such as visual processing (Eisenberg et al., 2018), meaning extraction in the visual domain (Strickland et al., 2015), and language (Malaia et al., 2021). Here, we ask whether there is primary research evidence for prediction in sign language processing in signing populations. After a systematic review grounded in PRISMA and PICOS frameworks, we identified studies that provided evidence for PP in signing populations across two linguistic levels (semantic, phonological) in multiple experimental paradigms, such as anticipatory eye gaze in the visual world paradigm (Lieberman et al., 2018; Wienholz and Lieberman, 2019) or N400 amplitude modulation in the semantic mismatch paradigm (Hosemann et al., 2013). However, investigations of motor simulation, hypothesized on the basis of predictive processing framework (Brozdowski, 2018; Brozdowski and Emmorey, 2020) found no evidence of motor simulation underlying PP in proficient signers. This does not, by itself, imply that no predictive processing takes place—rather, it indicates that predictive models do not appear to propagate to the level of motor simulation.

### Semantic Predictive Processing in Sign Languages

Our results indicate that semantic prediction has been the most researched so far. The reported studies provide evidence for the prediction of semantic information during continuous signing stream (Hosemann et al., 2013; Lieberman et al., 2018; Wienholz and Lieberman, 2019), as they did not use visually manipulated material (for the importance of naturalistic SL stimuli, see section “Relations Between Other Variables and Predictive Processing”). Findings that semantically constraining contexts enable semantic prediction in SLs align well with extensively studied spoken languages (Federmeier, 2007; Huettig, 2015; Kuperberg and Jaeger, 2016; Bornkessel-Schlesewsky and Schlesewsky, 2019).

### Other Types of Linguistic Predictive Processing

Brozdowski (2018) and Brozdowski and Emmorey (2020) investigated whether or not signers rely on the transitional movements between signs, hence whether they exploit phonological information to enable prediction, based on the motor simulation in forward models. However, they found no strong evidence for motor simulation. This suggests that PP does exist in sign language, as would be expected for all languages, but that motor simulation as a production account of PP, does not provide the best explanation for its underlying mechanism. Furthermore, Hosemann et al. (2013) employed a semantic violation paradigm, but they analyzed EEG data from different time points between the previous sign and the following critical sign. Thus, although they examined prediction in semantically constraining sentences, they were actually looking for phonological cues that could inform prediction by placing triggers in transitional movements. This aligns well with the Multiscale Information Transfer framework (Blumenthal-Dramé and Malaia, 2019), which emphasizes the interplay of multiple scales in SL processing.

As for other language-based variables that might affect PP, such as phonetic (articulatory) complexity, syntax, or frequency-based prediction, we did not find any research that addressed them. However, psycholinguistic properties of signs such as iconicity, frequency, or concreteness have been found to elicit distinct neurophysiological responses (Emmorey et al., 2020), suggesting differential processing. Thus, it would be worthwhile to explore the relationship between these psycholinguistic properties and PP in future studies.

### Relations Between Other Variables and Predictive Processing

#### Type of Stimuli

The stimuli from all the records included in the final synthesis were dynamic, which is not surprising given the nature of the dynamic, continuous sign language stream. This has become something of a standard in recent SL experimental research, compared to older studies. They used a sign-by-sign presentation due to the technical limitations of the time (e.g., Neville et al., 1997) or trimmed transitional movements between the critical sign and the rest of the sentence to avoid possible coarticulation effects and differences between conditions (Grosvald et al., 2012; Gutierrez et al., 2012). Nevertheless, it is important to have non-manipulated, naturalistic SL stimuli because there is experimental evidence for the role of transitional movements in semantic prediction (Hosemann et al., 2013) as well as in the resolution of ambiguous argument structures (Krebs et al., 2018), at least in sentential contexts. On the other hand, single-sign priming studies using clipped sign stimuli (i.e., videos were clipped to the onset of the sign, thus not showing transitional movements -cf. Gutierrez et al., 2012; Meade et al., 2018; Lee et al., 2019; Emmorey et al., 2022) report N400 as indicative of priming effects prior to the onset of the critical sign. However, due to the nature of the priming paradigm and the use of isolated signs, it is possible that the transitional movements did not turn out to be important for this very reason. Indeed, there is theoretical and experimental evidence for their importance at the sentence level. Namely, SLs are multilayered and signers process the visual properties of motion at multiple levels (Blumenthal-Dramé and Malaia, 2019). Moreover, transitional movements inform language comprehension, as noted above (Hosemann et al., 2013; Krebs et al., 2018).

#### Type of Task

In the studies by Brozdowski and Emmorey (2020), Experiment 1 and 2 by Brozdowski (2018), Study 1 by Brookshire et al. (2017) and Brookshire (2018), both Deaf signing and hearing non-signing participants performed the same task. This is understandable from the perspective of controlling for the effects of sign language dominance or the effects of long-term experience in the visual domain. Nevertheless, the nature of the task does not affect Deaf signers and hearing non-signers equally. For example, signers have enhanced spatial processing abilities (Emmorey, 2002; Pyers et al., 2010; Malaia and Wilbur, 2014), suggesting that these abilities might affect performance in the experiment and should be controlled for. In addition, signers imitate manual signs better than non-signers (for a review, see Rudner, 2018). Finally, hearing non-signers show different activation patterns in the sensorimotor cortex when perceiving signs than Deaf fluent signers (Kubicek and Quandt, 2019). Consequently, because hearing participants are unfamiliar with sign language and the perception of such complex visual stimuli, these results could be influenced by the increased cognitive load of observing such stimuli. The studies reviewed, involving both Deaf signing and hearing non-signing participants, did not report any measures of visual-spatial abilities or verbal working memory, with the exception of Brozdowski and Emmorey (2020), who developed a new test of motor memory. Nevertheless, they have not addressed the issue of different verbal working memory spans for spoken and sign language stimuli (Rudner, 2018; Malaia and Wilbur, 2019), although they acknowledge that motor memory and working memory are separate (Wu and Coulson, 2014). Overall, it is currently unclear whether other cognitive abilities had an impact on the performance of the non-signers from the above studies, and if so, to what extent.

#### Sign Language Competence

The data extracted for the target languages show that only unimodal language prediction was studied. Therefore, we cannot determine whether or not cross-modal prediction effects could be observed for bimodal bilinguals. This question should be addressed for three reasons. First, signers have been found to co-activate signs while reading (Morford et al., 2011; Meade et al., 2017; Villwock et al., 2021) as well as to co-activate written/spoken words while comprehending sign pairs (Lee et al., 2019) and sentences (Hosemann et al., 2020) and in the production of signs (Gimeno-Martínez et al., 2021). Second, there is evidence for cross-modal prediction for spoken languages (Sánchez-García et al., 2011, 2013). Third, the cross-modal prediction has also been found for other non-linguistic cognitive domains, such as perception of emotions (Jessen and Kotz, 2013) and music (Dercksen et al., 2021). Given this evidence for cross-modal interactions in both linguistic and non-linguistic domains, further studies might investigate whether bimodal bilinguals make cross-modal linguistic predictions.

Regarding the population parameters, all studies used either Deaf native/native-like L1 signers or hearing non-signers. Therefore, it is not clear at this moment whether different levels of SL dominance (such as L1 vs. L2 vs. bimodal bilingual) interact with the neural bases of prediction. However, it is reasonable to expect such differences in the manual-visual modality for two reasons. First, these effects have been found in studies of PP for spoken languages in cases of L1 vs. L2 groups (Martin et al., 2013; Kaan, 2014; Hopp and Lemmerth, 2018; Chun and Kaan, 2019; Schlenter, 2019; Henry et al., 2020). Second, differences in SL processing in other linguistic domains have been found to be a function of the SL age of acquisition (Malaia et al., 2020; Krebs et al., 2021).

### Suitability of Predictive Processing Models to Sign Language Data

Various models have been developed that attempt to explain linguistic prediction. As mentioned in section “Models of Predictive Processing,” most of them have been developed based on spoken language, with the exception of the Multiscale Information Transfer framework (Blumenthal-Dramé and Malaia, 2019), which specifically considers sign languages. However, from the studies we included in our systematic review, it appears that only production-based models have been tested so far, more specifically Pickering and Garrod’s (2007, 2013) prediction-by-production account. Hosemann et al. (2013) interpreted their findings in the context of a forward model. Based on the N400 amplitude modulation that started during the transitional movement before the critical lexical sign, they argue that signers recruited their forward models. Similarly, Brozdowski (2018) and Brozdowski and Emmorey (2020) originally hypothesized that signers engage in motor simulation, a mechanism thought to underlie linguistic prediction via production systems, but found insufficient evidence to support this model. However, as they note, it is possible that fluent signers do not use production systems for predictions during simple tasks. As suggested earlier in section “Sign Language Competence,” future studies should include signers with different levels of proficiency to elicit a variety of qualitative mechanism(s) for predictive processing (cf. Schlenter, 2019, for a similar treatment of proficiency in spoken languages, and underlying qualitative differences in predictive processing). Other studies eligible for this review reported that both children and adults made anticipatory gaze to the target item (Lieberman et al., 2018; Wienholz and Lieberman, 2019), but the authors did not discuss their findings in the context of a specific model of PP.

## Conclusion

In this paper, we have presented the results of the systematic review of studies on predictive processing (PP) in sign languages. We have also investigated the conditions under which it occurs. Our results show that most of the reviewed studies focused on semantic prediction. On the other hand, more recent studies have focused on the phonological basis of prediction during transitional movements between signs. However, there is currently no evidence for PP in other linguistic domains, such as frequency-based, phonetic (articulatory), and syntactic prediction. Regarding the conditions under which PP occurred, we found that semantic prediction has been studied mainly in adults and to a lesser extent in children (aged 4–8 years). Currently, the neural bases of PP in signing populations are inconclusive, as only three studies used EEG and no neuroimaging studies were found. The question of the mechanism of interaction between one’s sign language dominance (L1 vs. L2 vs. bimodal bilingual) and PP in the manual-visual modality is not clear, mainly because participants with different degrees of language dominance are missing. Altogether, the findings from SL studies, which corroborate findings from spoken language studies, suggest that PP is the modality-independent property of language processing, although the relatively small number of studies on PP in SLs limits our understanding of the modality-specific characteristics. Further studies are needed to improve our understanding of prediction in other linguistic domains in the visual-manual modality, e.g., syntax, morphology, pragmatics, as well as the interfaces between linguistic levels. In addition, the development of corpora from different SLs is needed to enable the extraction of linguistic measures from specific levels. Finally, the question of the underlying mechanism(s) of PP in relation to population parameters is relevant to the effects of age of acquisition on PP and whether it facilitates comprehension and/or production in SLs.

## Limitations

It is highly likely that publication bias has affected the availability of study information. By publication bias, we mean that studies with negative evidence (those that tested for a specific level/modality of PP and did not find statistically significant effects) were not published. This bias can be mitigated by the inclusion of doctoral dissertations (two included in the final study set) and registered reports (studies that pre-plan the assessment, and are accepted for publication prior to data collection, when analysis results are not known). However, the systematic search did not yield any registered reports in the domain.

## Data Availability Statement

The original contributions presented in the study are included in the article/supplementary material, further inquiries can be directed to the corresponding author.

## Author Contributions

TR and EM contributed to the design of the study. TR collected the data and wrote the first draft of the manuscript. All authors contributed to the revision of the manuscript and analyzed the data.

## Conflict of Interest

The authors declare that the research was conducted in the absence of any commercial or financial relationships that could be construed as a potential conflict of interest.

## Publisher’s Note

All claims expressed in this article are solely those of the authors and do not necessarily represent those of their affiliated organizations, or those of the publisher, the editors and the reviewers. Any product that may be evaluated in this article, or claim that may be made by its manufacturer, is not guaranteed or endorsed by the publisher.

## Appendix

### Search Strategy

We performed an advanced search in the databases listed in section “Data Sources and Search Strategy.” In the Scopus database, we used the following query: (KEY (“sign language”) OR KEY (“signed language”)) AND (KEY (prediction) OR KEY (anticipat*) OR KEY (forward) OR KEY (entrain*)) AND (EXCLUDE (SUBJAREA, “COMP”) OR EXCLUDE (SUBJAREA, “ENGI”)). Three exclusion filters were applied, so that records from the subject areas “Computer Science” and “Engineering” and non-English records were excluded.

Then, for the Web of Science database, we used the following sequence of terms: (AK = (“sign language” OR “signed language”)) AND AK = ((prediction) OR (forward) OR (entrain*) OR (anticipat*)), where AK stands for “author keywords.” We further refined the results by excluding the Web of Science category “Computer Science Artificial Intelligence.”

In ScienceDirect, we used the following sequence of terms under the section “Title, abstract or author-specified keywords”: (“sign language” OR “signed language”) AND ((prediction) OR (forward) OR (entrainment) OR (anticipatory)). In addition, the subject areas Computer Science, Engineering, Medicine and Dentistry, Energy, Material Sciences, Biochemistry, Genetics and Molecular Biology were excluded.

Next, the ProQuest database was searched for doctoral dissertations, with the following search query string: IF (“sign language” OR “signed language”) AND IF ((prediction) OR (forward) OR (entrain*) OR (anticipat*)), where IF stands for “identifier.” No exclusion filters were applied as there was only one result.

We searched APA PsycInfo database via EBSCOhost, using this search string for keywords: (“sign language” OR “signed language”) AND ((prediction) OR (forward) OR (entrain*) OR (anticipat*)). We did not apply any additional filters.

Finally, we conducted an advance search of the MEDLINE database, which was accessed through PubMed. The title and abstract fields were searched using the following string: (“sign language” OR “signed language”) AND ((prediction) OR (forward) OR (entrain*) OR (anticipat*)). No additional filters were applied.
